# A rare case report on esophageal squamous cell carcinoma metastatic to the colon

**DOI:** 10.3389/fonc.2026.1795201

**Published:** 2026-07-24

**Authors:** Lixia Hu, Yanyan Zha, Chengfa Li, Mengqin Huang, Qianqian Yuan, Yan Wu, Wei Cheng

**Affiliations:** Department of Oncology, The Second People’s Hospital of Hefei, Hefei, Anhui, China

**Keywords:** case report, colonic metastasis, esophageal squamous cell carcinoma, immunotherapy, rare metastasis

## Abstract

Esophageal squamous cell carcinoma(ESCC) carries a poor prognosis with low survival rates, frequently due to advanced disease at diagnosis. Colonic metastasis from ESCC is exceptionally rare. This report describes the case of a 68-year-old male with locally advanced ESCC who developed metachronous colonic metastasis during follow-up after initial definitive chemoradiotherapy combined with immunotherapy. Pathological examination of a colonic biopsy confirmed metastatic squamous cell carcinoma with an immunohistochemical profile consistent with esophageal origin. The patient subsequently received systemic chemotherapy followed by combined immunotherapy, achieving a partial response on imaging evaluation. This case not only underscores the rarity of colonic metastasis in ESCC but also highlights the critical importance of maintaining vigilance for this possibility within multidisciplinary care and long-term follow-up.

## Introduction

Esophageal cancer ranks as the seventh leading cause of cancer-related mortality globally (1). The predominant histological subtype worldwide is esophageal squamous cell carcinoma(ESCC) (2). Approximately 25% of patients with esophageal cancer present with distant metastases at initial diagnosis (3). Although treatment advances have improved survival rates for esophageal cancer patients, the outcomes are still poor, as evidenced by a 5-year overall survival rate of merely 10%–30% (4). The most common distant metastasis sites of ESCC are lymph nodes, liver and lungs (5). Unusual sites of esophageal cancer metastasis have been documented, including the cutaneous (6), patella (7) and rectal (8). This rarity may lead to insufficient clinical recognition, delayed diagnosis, or misdiagnosis. We report a rare case of colonic metastasis of ESCC.

## Case report

**Figure 1:**
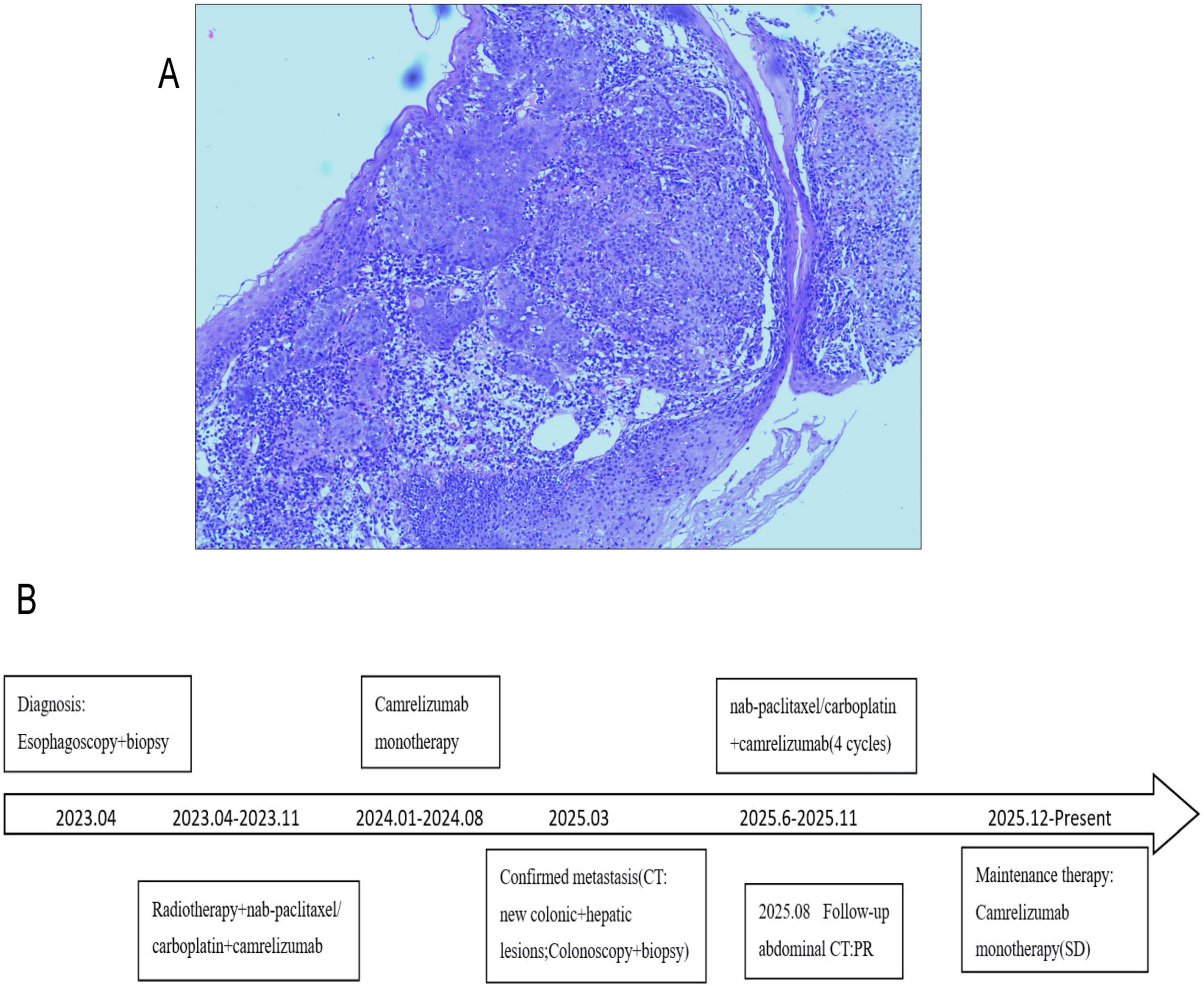
**(A)** Hematoxylin-eosin staining results of biopsy specimens of esophageal tumors(*100). **(B)** Timeline of treatments.

**Figure 2:**
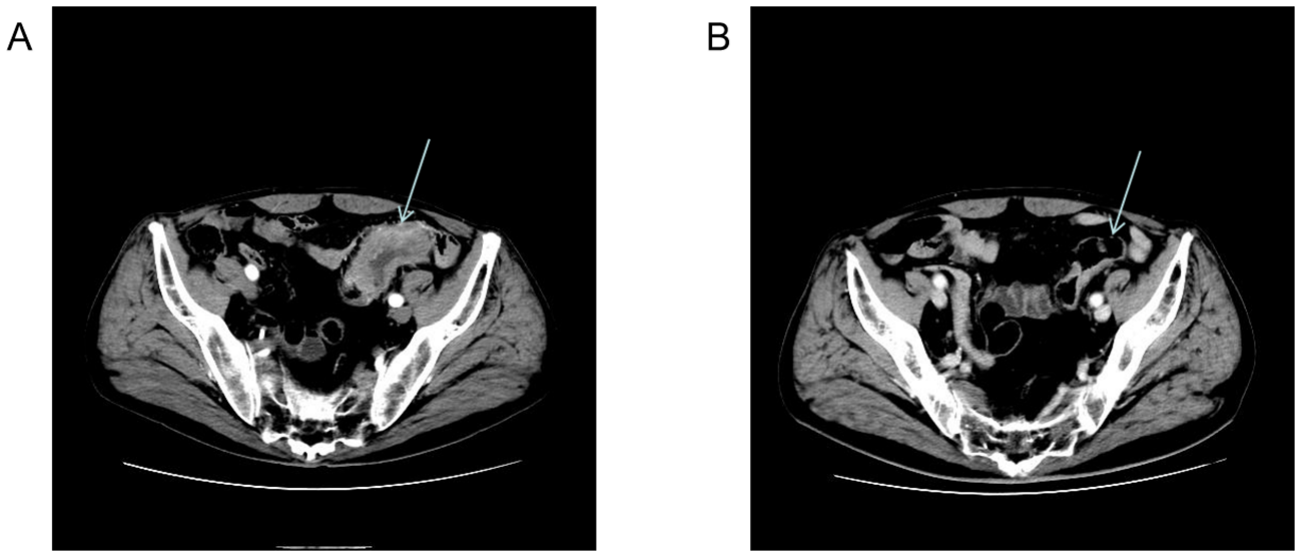
**(A)** CT demonstrated a space-occupying lesion in the left pelvic descending colon region; **(B)** CT imaging indicated a partial response according to the Response Evaluation Criteria In Solid Tumors(After two cycles of antitumor therapy).

**Figure 3:**
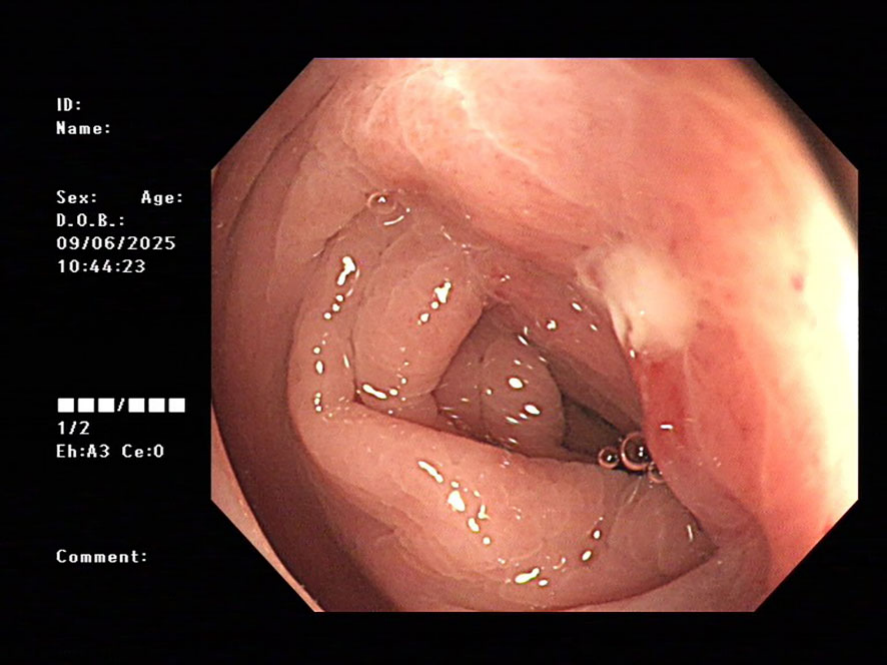
A colonoscopy revealed an ulcer measuring approximately 0.6cm*0.8cm located 20cm from the anal verge.

**Figure 4:**
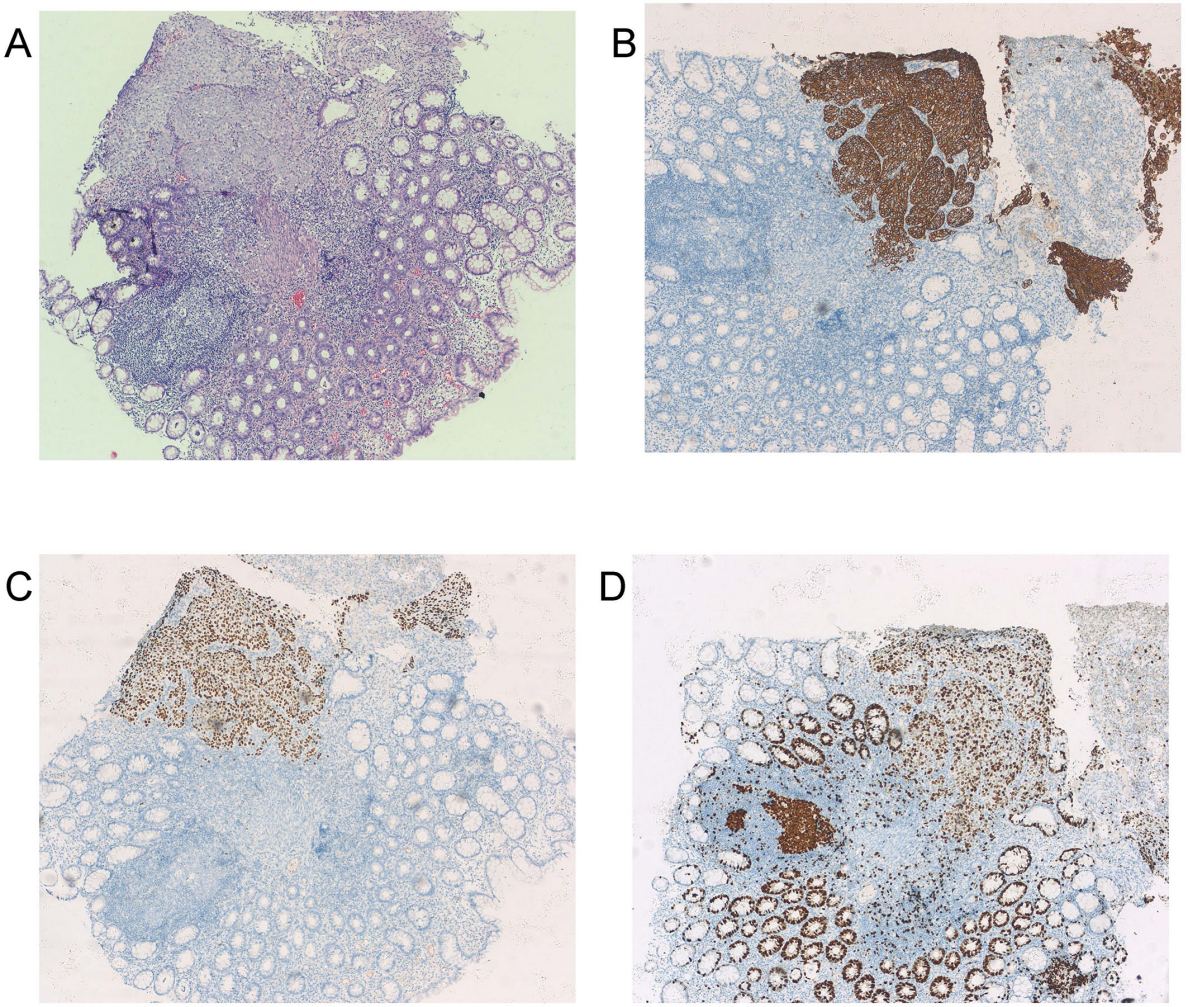
HE staining and immunohistochemical results supported squamous cell carcinoma **(A)** x100 (hematoxylin and eosin staining). **(B)** Positive CK5/6, **(C)** Positive p40, **(D)** positive Ki67. Magnification, x100.

A 68-year-old male presented in January 2023 with a 2-year history of progressive dysphagia. Esophagoscopic evaluation revealed a protruding lesion of the esophageal wall located 32-36cm from the incisors, occupying half of the esophageal circumference. Histopathological findings showed squamous cell carcinoma after biopsy (**Figure 1A**). PET/CT scan in April 2023 showed FDG-avid thickening of the lower thoracic esophagus and enlarged lymph nodes in the gastrohepatic region, consistent with locally advanced disease. Based on these findings, the patient was staged as cT3N1M0 (Stage III, AJCC 8th edition). Due to surgical contraindications, the patient received chemoradiotherapy combined with camrelizumab. Initially, from April to August 2023, camrelizumab was combined with albumin-bound paclitaxel and carboplatin for 4 cycles, concurrently with radical radiotherapy (60 Gy/30 fractions) to the primary esophageal lesion and involved lymph nodes. During this period, the patient developed severe myelosuppression and a febrile episode with pulmonary infection, which were managed supportively. This was followed by two additional cycles of camrelizumab plus chemotherapy in October and November 2023, after which the patient received maintenance camrelizumab monotherapy from January to August 2024 (approximately 9 months). The disease remained stable throughout this period. Enhanced Scan (Whole Abdomen and Pelvis Continuous Scan) Examination Report (March 19, 2025): (1) A space-occupying lesion is observed in the left pelvic descending colon region, suspected to be cancerous (**Figure 2A**). (2) A space-occupying lesion is noted at the edge of the right lobe of the liver, with unclear boundaries from the adjacent diaphragm, suspected to be metastatic. Serum tumor markers, including CEA, CA19-9, and SCC-Ag, were within normal limits at this time. A colonoscopy revealed an ulcer measuring approximately 0.6cm*0.8cm located 20cm from the anal verge, and a biopsy was subsequently performed (**Figure 3**). Immunohistochemistry results were as follows:CK7(-)、CEA(-)、CK20(-)、CK5/6(+)、P40(+)、Ki-67(+, approximately 70%) (**Figure 4**). Cell morphology and immunolabeling results confirmed that the esophagus was the origin. Salvage therapy was then initiated with camrelizumab combined with albumin-bound paclitaxel and carboplatin, administered on June 24, July 23, October 13, and November 11, 2025 (4 cycles total). Follow-up abdominal CT in August 2025 showed slight reduction in the size of the hepatic and perinephric lesions and improved colonic wall thickening, indicating a partial response (**Figure 2B**). Post-chemotherapy fever occurred and was managed with anti-infective treatment. Following these four cycles, the patient achieved stable disease and has continued on camrelizumab maintenance therapy from December 2025 to the present, with no evidence of disease progression. Regarding safety, the main adverse event was myelosuppression (grade 3 neutropenia managed with G-CSF), and no severe immune-related adverse events such as pneumonitis, colitis, or hepatitis occurred throughout the entire treatment course. The patient received camrelizumab (200 mg IV, Q3W) across multiple phases throughout the treatment course. The treatment timeline was shown in **Figure 1B**.

## Discussion

The incidence and mortality of ESCC continue to be elevated in several countries, most notably in China (9). Owing to its deep-seated location and clinically silent progression, most esophageal cancer patients present with locally advanced or advanced disease at diagnosis. We describe a case of ESCC in which a colonic mass was detected during follow-up. The clinical question was whether this represented a metastasis from the ESCC or a separate primary malignancy of the colon.

However, colorectal metastasis from ESCC remains exceptionally rare, with fewer than 20 reported cases in the English-language literature to date. Kang et al. (10) reported a case of a 63-year-old male with ESCC who developed an isolated sigmoid colon metastasis, which was initially detected by abnormal ¹^8^F-FDG uptake on PET/CT and subsequently confirmed by histopathological examination. Yang et al. (11) reported a rare case of synchronous colonic and rectal metastases from ESCC in a 65-year-old male, who developed hematochezia and malignant bowel obstruction during follow-up after receiving chemotherapy combined with immunotherapy and radiotherapy. Garg et al. (12) reported a case of metachronous colonic metastasis from ESCC in a 60-year-old male with a history of laryngeal SCC, who developed a 3cm ascending colon mass approximately one year after completing chemoradiotherapy for mid-esophageal SCC; immunohistochemistry (CK5/6+, p63 weakly positive, CK20–, CDX2–) confirmed the diagnosis of metastatic SCC from esophageal origin. The patient received palliative radiation to the colonic lesion and died approximately 6 months after the diagnosis of metastasis. Wiseman et al. (13) described a 71-year-old woman who developed rectosigmoid metastasis 2 years after neoadjuvant chemoradiation and esophagectomy; palliative radiotherapy was administered, and she died 6 months later. Chen et al. (14) reported a case of a 68-year-old man with ESCC who initially received concurrent chemoradiotherapy. Six months after treatment, a follow-up ¹^8^F-FDG PET/CT revealed new hypermetabolic lesions in the colon and peritoneum, which were subsequently confirmed as metastatic squamous cell carcinoma by histopathological examination. Shimada et al. (15) reported a case of a 64-year-old male with middle thoracic ESCC who achieved stable disease after chemoradiotherapy. Preoperative colonoscopy incidentally revealed a 1.0cm submucosal tumor at the splenic flexure, which was pathologically confirmed as a metastasis from primary ESCC after concurrent resection. Uotani et al. (16) reported a case of a 67-year-old male with middle thoracic esophageal squamous cell carcinoma who received neoadjuvant chemotherapy followed by subtotal esophagectomy. Approximately 1 year after esophagectomy, an asymptomatic, solitary 5cm mass at the ascending colon was detected on routine follow-up CT and confirmed as metastasis from primary ESCC by biopsy. Additionally, Zhang et al. (17) reported the first case of esophageal squamous cell carcinoma metastasizing to a colonic anastomotic site in a 73-year-old woman with a history of right hemicolectomy 14 years earlier. She presented with dysphagia and was found to have a 7cm esophageal mass and a 7mm ulceration at the ileocolonic anastomosis, both biopsied as moderately to poorly differentiated squamous cell carcinoma with identical morphology and immunoprofile, confirming metastasis from esophageal origin.

Immunohistochemistry plays an important role in determining the origin of the primary lesion of metastatic tumors. Due to its rarity, ESCC colonic metastasis lacks characteristic clinical presentations or specific imaging signs. Patients may present with symptoms such as abdominal pain, altered bowel habits, obstruction, or bleeding, which are also common in primary colon cancer or other abdominal pathologies (18). Therefore, pathological diagnosis is crucial and definitive. As demonstrated in this case, endoscopic biopsy coupled with a systematic immunohistochemical panel is the gold standard for confirmation. The differential diagnosis of a colonic lesion in a patient with a history of esophageal squamous cell carcinoma encompasses several possibilities: (1) metastatic squamous cell carcinoma from the primary esophageal tumor; (2) a synchronous primary colorectal adenocarcinoma; (3) primary colorectal squamous cell carcinoma, an exceedingly rare entity; and (4) metastasis from another occult primary squamous cell carcinoma. The distinction of ESCC from primary colonic squamous cell carcinoma (an exceptionally rare entity) or adenocarcinoma relies heavily on its characteristic immunoprofile, which typically includes positivity for CK5/6 and P40, and negativity for markers such as CK7 and CK20. In this case, the colonic lesion demonstrated this characteristic immunoprofile (CK5/6+, P40+; CK7−, CK20−, CEA−), which definitively excludes primary colorectal adenocarcinoma (typically CK20+, P40−) and argues against metastasis from lung or cervical primaries (frequently CK7+). Primary colorectal squamous cell carcinoma, although sharing a similar immunophenotype, is exceptionally rare and would not explain the synchronous presence of the esophageal primary with identical morphology. Emphasizing the experience of pathologists and ensuring thorough communication between the pathology department and clinicians are vital to avoid misdiagnosis as primary colon cancer. The Ki-67 proliferation index in the colonic metastasis was approximately 70%, indicating aggressive tumor biology and correlating with disease progression during maintenance therapy. Notably, despite this high proliferative activity, the tumor achieved a partial response to subsequent salvage immunochemotherapy, suggesting that a high Ki-67 index does not predict resistance to immunotherapy.

The occurrence of colonic metastasis from ESCC is exceedingly rare, with potential mechanisms including lymphatic dissemination and hematogenous spread (19). The hypothesized mechanism of lymphatic metastasis suggests that due to the dense lymphatic network within the esophageal wall, tumor cells could theoretically undergo retrograde transport via lymphatic vessels to the colorectum and establish metastases (11, 20). In addition, this case demonstrates a distinct “esophagus → liver → colon” route of hematogenous metastasis. Venous drainage from the mid-to-lower esophagus converges into the portal vein via vessels such as the left gastric vein, explaining the concurrent discovery of a metastatic lesion in the right hepatic lobe, which provides crucial “way-station” evidence for hematogenous spread (21). Subsequently, tumor cells from the hepatic metastasis can detach, enter the systemic circulation via the hepatic veins, and disseminate to the colon through the inferior mesenteric arterial system (22). This pathway perfectly accounts for the localization of the metastasis within the sigmoid colon mucosa, consistent with the colonization pattern of hematogenous spread.

Immune checkpoint inhibitors (ICIs) have become a standard of care for recurrent or metastatic (23, 24). The management of this case exemplifies the principles of multidisciplinary comprehensive care for advanced ESCC. Initial therapy employed a combined regimen based on the immune checkpoint inhibitor camrelizumab. Following the detection of a rare metastatic site, the treatment strategy was promptly adjusted, continuing with combined immunotherapy and chemotherapy, which demonstrated a favorable short-term response. This suggests that even in the context of rare metastases, systemic therapy based on the primary tumor’s biological characteristics remains an effective cornerstone.

Nevertheless, as a single case report with limited tissue samples precluding genomic analysis, our findings require validation in larger cohorts. Future studies incorporating molecular profiling are needed to better clarify the mechanisms and optimal management of this rare metastatic pattern.

## Conclusion

This case report details an exceptionally rare instance of colonic metastasis from ESCC. It is crucial to perform a biopsy and histological analysis on such incidentally detected lesions, as this helps differentiate between metastatic and primary cancer. The goal of multidisciplinary comprehensive treatment is to implement palliative therapy to improve the quality of life and prolong survival in patients with such metastatic disease.

## Data Availability

The original contributions presented in the study are included in the article/supplementary material. Further inquiries can be directed to the corresponding author.
